# Make Yourself Useful

**Published:** 1869-12

**Authors:** 


### "MAKE YOURSELF USEFUL."

THIS is the standing injunction to a large family of steady and affectionate children, of a "Friend" mother, whom we know, and we regard it as one of the most important lessons which childhood can learn. Many a young man would have been saved from the halter, had he learned in his father's house how to "make himself useful," under all the circumstances of life. And well do we know, that many a girl with a good heart, has gone down to an early grave of infamy, from being brought up, by a false kindness, without the knowledge of how she was to "make herself useful" in the various changes and adversities of life. Very many girls, in this drear winter weather, go to bed hungry, and rise, to hover around stinted fires, and shiver all day in scanty clothing, wearing the wrinkles of sadness and care on faces yet in their teens, willing enough to work—and abundant work to do, with liberal pay, in luxurious mansions, whose rich occupants would count it a "fortunate thing," to find a person suited to the place.

Why then, are the doors of all our charities beseiged and daily thronged? and why does the pitiful appeal strike the ear of the pedestrian in his early walk, or noon-day promenade, or nightly visit to the party, the lecture, the concert, or the opera? It is simply because these starving, freezing girls were not brought up to be useful, were not taught, by careless or over-indulgent mothers, how they might command situations. The incessant and earnest cry of thousands of almost despairing housekeepers, is for competent "help," to cook, to nurse, to sew; for chamber work, or for waiting. Within any twenty-four hours, two thousand, may we not say, ten thousand, such girls could find welcome homes, in the very best families in New York, at high wages.

But American girls think it degrading to cook, and nurse, and wash, and wait on the table, and their more inexcusable and short sighted parents confirm them in their views; and the next we hear of them, is "starvation," "suicide," premature disease, or a dishonored grave. Let all these, especially those who can leave their families nothing, impress on the minds of their children day by day, that it is more dishonorable to beg than to work; that it is more criminal to do nothing, than to be industrious; that no employment is dishonorable, which is useful; and that it is not only a disgrace but a crime, to be idle, from feelings of a despicable false pride.

